# Genetically Encoded Photosensitizers as Light-Triggered Antimicrobial Agents

**DOI:** 10.3390/ijms20184608

**Published:** 2019-09-17

**Authors:** Fabienne Hilgers, Nora Lisa Bitzenhofer, Yannic Ackermann, Alina Burmeister, Alexander Grünberger, Karl-Erich Jaeger, Thomas Drepper

**Affiliations:** 1 Institute of Molecular Enzyme Technology, Heinrich-Heine-University Düsseldorf, Forschungszentrum Jülich GmbH, D-52428 Jülich, Germany; f.hilgers@fz-juelich.de (F.H.); n.bitzenhofer@fz-juelich.de (N.L.B.); yannic.ackermann@uni-duesseldorf.de (Y.A.); k.-e.jaeger@fz-juelich.de (K.-E.J.); 2 Multiscale Bioengineering, Bielefeld University, D-33501 Bielefeld, Germany; a.burmeister@fz-juelich.de (A.B.); alexander.gruenberger@uni-bielefeld.de (A.G.); 3 Institute of Bio- and Geosciences, IBG-1: Biotechnology, Forschungszentrum Jülich GmbH, D-52428 Jülich, Germany

**Keywords:** photosensitizer (PS), light-oxygen-voltage (LOV) proteins, antimicrobial photodynamic inactivation (aPDI), green fluorescent protein (GFP), flavin-binding fluorescent protein (FbFP), optogenetics, extracellular phototoxicity, antibiotics

## Abstract

Diseases caused by multi-drug resistant pathogens have become a global concern. Therefore, new approaches suitable for treating these bacteria are urgently needed. In this study, we analyzed genetically encoded photosensitizers (PS) related to the green fluorescent protein (GFP) or light-oxygen-voltage (LOV) photoreceptors for their exogenous applicability as light-triggered antimicrobial agents. Depending on their specific photophysical properties and photochemistry, these PSs can produce different toxic ROS (reactive oxygen species) such as O_2_^•−^ and H_2_O_2_ via type-I, as well as ^1^O_2_ via type-II reaction in response to light. By using cell viability assays and microfluidics, we could demonstrate differences in the intracellular and extracellular phototoxicity of the applied PS. While intracellular expression and exogenous supply of GFP-related PSs resulted in a slow inactivation of *E. coli* and pathogenic Gram-negative and Gram-positive bacteria, illumination of LOV-based PSs such as the singlet oxygen photosensitizing protein SOPP3 resulted in a fast and homogeneous killing of these microbes. Furthermore, our data indicate that the ROS type and yield as well as the localization of the applied PS protein can strongly influence the antibacterial spectrum and efficacy. These findings open up new opportunities for photodynamic inactivation of pathogenic bacteria.

## 1. Introduction

Since the rapid worldwide emergence of multi-drug resistant bacteria, in conjunction with a decline in the development and production of new antimicrobial agents, the efficient treatment of various life-threatening pathogens has become increasingly endangered. For this reason, major research efforts aim to develop alternative antimicrobial therapies to prevent, treat, and finally eliminate multidrug resistance [1,2,3]. Antimicrobial photodynamic inactivation (aPDI) evolved in the last years as a method to treat microbial infections after realizing the potential of photodynamic therapy (PDT), which is increasingly used in cancer therapy [4,5]. PDT and aPDI combine the use of visible light with a light-sensitive dye—referred to as photosensitizer (PS)—and are based on the local formation of toxic reactive oxygen species (ROS). The produced ROS react fast with molecules of the PS microenvironment and thus can immediately induce damages of lipid membranes, cell walls, proteins, and nucleic acids [6,7,8]. Because of the broad spectrum of ROS-sensitive targets, aPDI does not induce resistances in microorganisms and further allows efficient inactivation of multi-drug resistant pathogens [9,10,11].

Upon light absorption, the PS undergoes a transition from the electronic ground state to a singlet excited state and further to a longer-lived triplet state via intersystem crossing (ISC). Here, the generation of ROS can follow two alternative pathways: the triplet state-PS can interact with molecular oxygen by transferring an electron to O_2_ yielding a superoxide radical anion (O_2_^•−^) that can further be converted into other ROS, including hydrogen peroxide (H_2_O_2_) and the hydroxyl radical (HO^•^). This pathway is referred to as a type-I mechanism. Alternatively, the type-II pathway involves an energy transfer from the excited PS to molecular oxygen, thereby generating singlet oxygen (^1^O_2_). Due to its unstable electron configuration, this form is extremely transient and highly reactive, resulting in a lifetime of up to 2 µs and a diffusion range of ~150 nm, depending on the dynamics of the photosensitizing protein [12,13]. In contrast, hydrogen peroxide shows a lifetime of about 1 ms and thus can diffuse over longer distances or even between microbial cells [14,15].

Because of the short ROS lifetimes, the localization of the applied PSs and their close proximity to microbial target molecules can play an important role for efficient aPDI. Therefore, cationic photosensitizing chromophores are frequently used to predominantly bind negatively charged surfaces of Gram-positive and Gram-negative bacteria, thereby avoiding excessive damage to mammalian cells and tissues [16,17]. Most widely used cationic PSs, whose antibacterial activities against multi-drug resistant pathogenic bacteria could already successfully be demonstrated, include porphyrinoids like porphyrins, chlorins, and phthalocyanines, as well as fullerenes and phenothiazinium dyes (e.g., toluidine blue O and methylene blue) [18,19,20,21,22]. However, the application of chemical PSs as light-triggered anti-infectives face some major drawbacks, including (i) a limited selectivity for bacteria and pathogens, (ii) an inefficient uptake by microbial cells, (iii) their subsequent secretion by microbial multidrug efflux pumps, and (iv) their heterogeneous distribution within a microbial population or biofilm. In addition, the local environment can strongly influence the photophysics of a PS, which might result in a divergent phototoxicity in dependence on its localization and the targeted pathogen. These limitations provoked the development of more effective PSs including genetically encoded PSs. In contrast to chemical PSs, genetic engineering approaches enable the fusion of tailored targeting sequences (e.g., leader peptides or antibodies) to photosensitizing proteins thereby facilitating their accumulation at particular cellular structures, compartments, or pathogens of interest. In addition, genetically encoded PSs can be seen as protein encased phototoxic chromophores where the protein envelope ensures a constant local environment and robust ROS formation irrespective of the PS localization [23].

Two major classes of genetically encoded PSs have been established. The first class includes fluorescent proteins (FPs), which exhibit a green fluorescent protein (GFP)-like structure; the second class encompasses flavin-binding fluorescent proteins derived from the light-oxygen-voltage (LOV) photoreceptor domain of plants, algae, and bacteria (LOV-PSs) [24,25,26,27]. KillerRed and the Singlet Oxygen Generator (miniSOG) were the first members of the GFP and LOV families that have been described as genetically encoded PSs [28,29]. So far, these photosensitizing proteins could successfully be applied, for example, (i) in the analysis of ROS signaling [30,31], (ii) for killing cancer cells in different PDT approaches [32,33,34,35,36], and (iii) for light-mediated control of protein activity via chromophore-assisted light inactivation (CALI) [37,38,39,40]. Recently, the KillerRed-derivatives SuperNova and KillerOrange as well as the miniSOG variant SOPP3 were engineered, showing improved photosensitizer properties [25,26,41]. While SuperNova has similar spectral characteristics as described for the original KillerRed protein with an absorption maximum at 579 nm, the spectrally tuned derivative KillerOrange exhibits a blue-shifted spectrum with absorption maxima at 455 and 514 nm [25,26]. In contrast, as a typical member of the LOV family, SOPP3 can be excited with blue light (λ_max_ = 440 nm, [41]). Besides their spectral characteristics, the three PSs differ significantly in their ability to form ROS when irradiated [23,42]. In comparison to all of the so far characterized LOV-based PSs, SOPP3 exhibits the highest singlet oxygen quantum yield (Φ_Δ_) of about 0.6 and spectroscopic in vitro characterization revealed that this photosensitizer protein selectively produces singlet oxygen via type-II reaction [41]. On the other hand, KillerRed—and presumably also its derivatives—primarily generate the superoxide anion and downstream oxidants, such as hydrogen peroxide through type-I photochemistry [42,43,44].

In a recent study, we demonstrated that most LOV-based fluorescent proteins, which were originally designed as alternative reporters for the in vivo analysis of oxygen-limited systems [45,46], were potent photosensitizers that could be applied for a light-controlled killing of *E. coli* when expressed intracellularly [47]. Here, we have evaluated the intracellular phototoxicity of three further GFP- and LOV-related PSs using *E. coli* as a model organism. In addition, we analyzed the antimicrobial efficacy and spectrum of exogenously applied GFP- and LOV-PSs with different photosensitizing activities towards Gram-positive and Gram-negative pathogens. Finally, we show data indicating that the cell envelope of the human pathogen *Pseudomonas aeruginosa* can be targeted by using the lectin LecB fused to the recombinant photosensitizing protein DsFbFP M49I, which resulted in an increased phototoxicity.

## 2. Results and Discussion

### 2.1. Phototoxicity of SOPP3, SuperNova, and KillerOrange in the Cytoplasm of E. coli 

**Figure 1:**
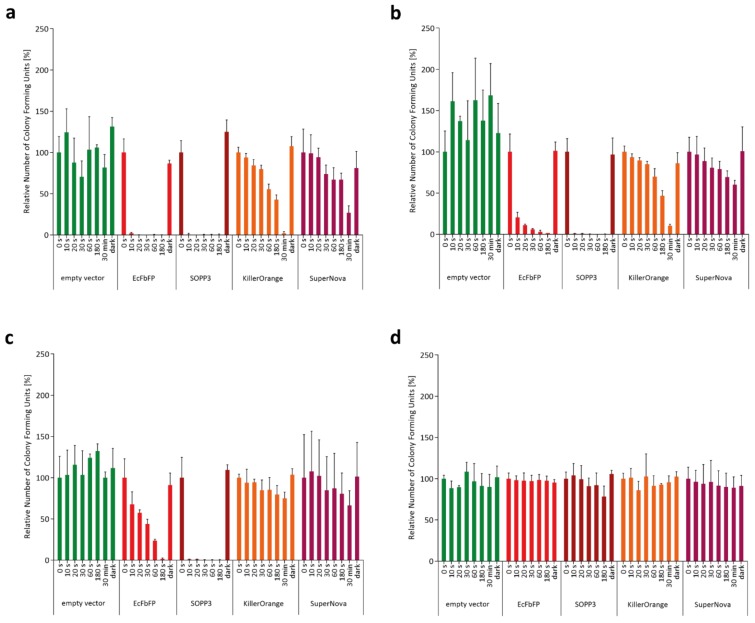
Analysis of colony forming units (CFU) for comparative analysis of in vivo phototoxicity of genetically encoded photosensitizers (PSs). The colony forming capacity of PS-producing *E. coli* cells was measured in dependence on illumination time and light intensity ((**a**) ~130 mW cm^−2^; (**b**) ~90 mW cm^−2^; (**c**) ~10 mW cm^−2^; and (**d**) ~1 mW cm^−2^). For this, cultures of *E. coli* BL21(DE3) cells harboring the respective expression vectors were diluted to a finale OD_580 nm_ of 0.1 in 1x PBS buffer (pH 7.4) and then illuminated with blue light (λ_max_ = 450 nm) and, in the case of SuperNova, with orange light (λ_max_ = 600 nm). As a control experiment, *E. coli* cells harboring an empty vector were also illuminated with blue light using the four light intensities. After given time points (0 to 30 min), aliquots of the irradiated cells were transferred to Lysogeny Broth (LB) agar plates and incubated overnight at 37 °C in the dark. Decrease of CFUs represents the time-dependent efficacy of the genetically encoded PSs. Data represent mean values of three independent experiments and their corresponding standard deviations indicated by error bars.

To compare the applicability of SOPP3, SuperNova, and KillerOrange for aPDI, we initially analyzed their intracellular phototoxicity. To this end, we determined the viability of PS-producing *E. coli* cells after illumination with blue light (LED with λ_max_ = 448 nm for SOPP3 and KillerOrange) or orange light (λ_max_ = 600 nm for SuperNova) by counting the colony forming units (CFU). The phototoxic effects of the endogenous PSs towards *E. coli* cells were measured in dependence on different light intensities (130–1 mW cm^−2^) as well as illumination times (0–30 min). As a reference, we additionally analyzed *E. coli* cells expressing EcFbFP, a LOV-based PS that was shown to perform moderate type-I and -II-mediated ROS formation resulting in an intermediate phototoxicity [47]. As shown in Figure 1, the increase of light intensity or illumination time resulted in a clear decrease of the number of viable bacterial cells for all of the tested LOV- and GFP-PSs, although the phototoxic efficacy differs strongly between the variants. 

Remarkably, upon illumination with light intensities of 130 to 10 mW cm^−2^, SOPP3 showed a very high phototoxicity as reflected by an almost complete cell death within the first 10 s of blue light illumination (Figure 1a–c). In comparison, for EcFbFP a more pronounced dependency on illumination time and light intensity could be observed. Surprisingly, the GFP-like PSs KillerOrange and SuperNova exhibited comparatively low light-induced toxicities. Consequently, high light intensities and prolonged illumination times up to 30 min were necessary to induce detectable cell death whereas low light intensities or short illumination times resulted in only minor or even no phototoxic effects (Figure 1). In contrast, for none of the applied illumination conditions significantly reduced cell viabilities could be observed when *E. coli* cells were used that harbor the empty expression vector. Furthermore, a very low light intensity of 1 mW cm^−2^ was not sufficient to induce an observable phototoxic effect for each of the tested PS proteins (Figure 1d). These control experiments clearly demonstrate that *E. coli* viability is only affected by appropriately illuminated PSs. Accordingly, the wavelength that has been used for the excitation of SOPP3 and KillerOrange did not activate phototoxicity of SuperNova and vice versa (Supplementary Figure S1a). Thus, the combination of blue light activated PSs with SuperNova allows a simultaneous application in one experimental setup, e.g., for studying the function of defined species within microbial consortia. 

**Figure 2:**
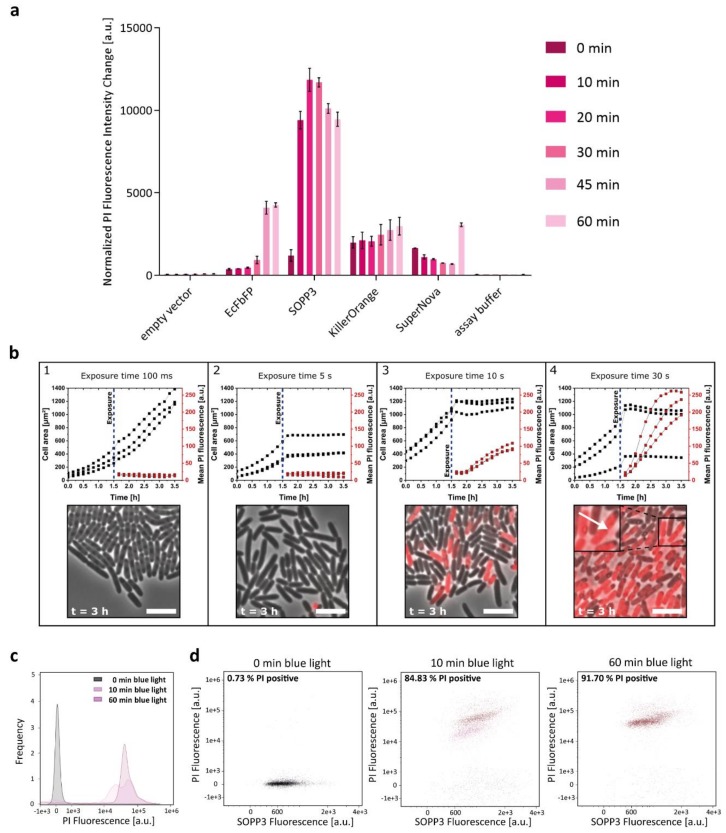
Quantitative in vivo phototoxicity studies of genetically encoded PSs using the propidium iodide (PI) cell death assay. (**a**) For the PI-based cell death assay, *E. coli* cells carrying the respective pET28a(+) derivatives were adjusted to an OD_580 nm_ of 0.5 in 1x PI assay buffer (pH 7.4) and illuminated with low light intensities (~10 mW cm^−2^) of blue (λ_max_ = 447 nm) or orange light (λ_max_ = 600 nm). The bars indicate the change in PI fluorescence intensity (λ_ex_ = 535 nm; λ_em_ = 617 nm) in dependence on exposure time. The data were normalized to the amount of functional protein per cell to exclude an influence of different protein accumulation levels. The data represent the mean values of three independent experiments and the error bars indicate the calculated standard deviations. (**b**) Intracellular phototoxicity of SOPP3 in *E. coli*. Growth and PI fluorescence data of single cells were monitored over time in microfluidic experiments using *E. coli* pET28a-SOPP3. (1) 100 ms blue light exposure (λ_max_ = 445 nm) after 1.5 h cultivation time. (2) 5 s blue light exposure after 1.5 h cultivation time. (3) 10 s blue light exposure after 1.5 h cultivation time (4) 30 s blue light exposure after 1.5 h cultivation time. The arrow indicates a position where a cell lysis event resulted in the release of red fluorescent nucleic acids. In each graph, data from three representative microfluidic chambers are shown. Scale bar = 5 µm. (**c**) Measurement of cell death by PI uptake via flow cytometry (FCM). *E. coli* cells harboring the expression vector pET28a-SOPP3 were analyzed for fluorescence and gated based on forward scatter (FSC) and side scatter (SSC) to exclude cell debris and accumulation of cells. Samples for FCM were prepared as described for the PI-based cell death assay. The propidium iodide fluorescence intensity of each cell was measured using a 561 nm-laser (and a 611/31 nm (red) bandpass filter) and plotted using a log scale. The data shown in the histogram represent the frequency of cellular PI signal intensities for SOPP3 producing *E. coli* cells before (0 min) and after 10 and 60 min of blue light illumination. (**d**) For quantitative analysis of ROS-mediated cell death, the SOPP3 fluorescence of *E. coli* cells was analyzed with a 488 nm-laser, detected by a 528/46 nm bandpass filter and plotted against the PI fluorescence before and after blue light illumination. Dead *E. coli* cells (presented as red populations) are shifted to higher PI fluorescence values and the percentage of dead cells is displayed in the upper left corner. Living cells are represented as black populations. An empty vector control was also analyzed to exclude a toxic blue light effect on the cells (Supplementary Figure S2c).

Since accumulation levels of PS proteins can strongly differ as demonstrated recently [47], we analyzed the intracellular phototoxicity of the chosen PS proteins in more detail by correlating cell death with the amount of functional photosensitizing protein per cell. To this end, we used the propidium iodide (PI) assay. The increase in PI fluorescence indicates the ability of the tested PS to damage *E. coli* cells through light-induced ROS generation. The obtained PI fluorescence can subsequently be normalized to the individual PS-dependent fluorescence intensity of cell culture as well as the specific fluorescence brightness of the respective PS and changes of PI fluorescence during the first 10-min irradiation period were used for comparing individual intracellular phototoxicities as described recently [47]. Due to the high phototoxicity of the LOV-based photosensitizers, these studies were performed at a lower light intensity (10 mW cm^−2^) to precisely determine illumination-dependent changes of *E. coli* cell viability in dependence on the corresponding PS activities. The results of the quantitative phototoxicity assay are presented in Figure 2a. As already indicated by the cell viability assay (Figure 1), SOPP3 exhibits by far the highest light-triggered antimicrobial activity of all tested LOV-based PSs as indicated by the almost maximally achievable change of PI fluorescence already after ten minutes of illumination (for comparison see also the results published by Endres and coworkers [47]). In contrast, the GFP-like proteins, KillerOrange and SuperNova are less-toxic photosensitizing proteins exhibiting no significant changes in PI fluorescence and only a slow and marginal increase of PI fluorescence, even after prolonged irradiation (Figure 2a) or increased light intensities (Supplementary Figure S1b).

Local variations in the cellular microenvironment including the uneven distribution of oxygen or nutrients, e.g., within a bacterial colony, can result in variations of cellular physiology and replication. Consequently, such inhomogeneities could affect the efficacy of PS-mediated phototoxicity that might, in turn, result in a delayed or heterogeneous killing of individual cells within a population. In a next step, we therefore analyzed if the light-induced antimicrobial effect occurs homogenously for every single cell on the microcolony level. For this purpose, *E. coli* cells expressing SOPP3, which showed the highest phototoxicity in previous experiments, were cultivated in microfluidic monolayer cultivation chambers. The growth chambers are designed to follow (i) cell growth, as well as (ii) distribution of intracellular protein accumulation (SOPP3 fluorescence), and (iii) cell death (PI fluorescence) before and after blue light exposure within a developing single-layered microcolony (Figure 2b and Supplementary Figure S2a).

The results of the representative microfluidic cultivation experiments are plotted as cell growth (chamber area that is occupied by growing cells) and mean PI fluorescence within a chamber during cultivation for four different exposure times (100 ms, 5 s, 10 s, and 30 s). Exemplary images of the cultivations at different exposure times are shown after 3 h cultivation. Interestingly, a strong impact of the exposure time on both the cell growth and the PI fluorescence intensity could be seen, leading to three different phenotypes. After an exposure time of 100 ms, cell viability is almost unaffected as indicated by the rapid increase of the cell area, while the respective PI fluorescence remained low. After an exposure of 5 s, the cell area stayed constant and therefore cell growth seemed to be impaired. However, no PI fluorescence signal was detectable; apparently, the cell membranes were not harmed yet by the produced ROS. Finally, after an exposure time of 10 s and 30 s, cell growth was completely inhibited and additionally, the PI fluorescence increased rapidly indicating a high ROS production and thereby an extensive damage of the cells and their membranes. Remarkably, although SOPP3 fluorescence was almost uniformly distributed in all cells as expected for the chosen *E. coli* expression system [48], a heterogeneous development of PI fluorescence could be observed after 10 s of blue light exposure which is independent of the respective position of the cells within the microcolony (Figure 2b and Supplementary Figure S2a). However, an illumination of 30 s resulted in an equally distributed PI fluorescence with comparable fluorescence signal intensities in all cells of the chamber, confirming a homogeneous and position-independent ROS response within the entire culture after prolonged illumination. 

To further analyze the distribution of PS toxicity within a larger population (10,000 cells), ROS-mediated cell damage was quantified via flow cytometry (FCM) (Figure 2c,d). Bacterial events were gated by size and granularity (FSC and SSC) (Supplementary Figure S2b) and then the bacterial population was analyzed regarding the PI and SOPP3 fluorescence. The PI fluorescence was plotted against the respective PS fluorescence before and after blue light illumination (exposure time: 10 and 60 min). As expected, no significant PI signal could be measured in cells that were kept in the dark as well as in *E. coli* cells harboring the empty vector (Supplementary Figure 2c). After 10 min of blue light exposure, roughly 85% of the analyzed cells are dead, as indicated by the positive PI signal (shown in red). Interestingly, two subpopulations with slightly differing PI fluorescence intensities could be observed after 10 min of blue light exposure (Figure 2d). However, longer blue light illumination resulted in comparably strong PI fluorescence in 92% of the analyzed cells (Figure 2d, 60 min). These results are in good accordance with the microfluidic data, as PS-mediated toxicity resulted in a “two step” induction of PI fluorescence in dependence on the applied illumination time. At this point, however, it is worth mentioning that even a low PI fluorescence signal of a single cell already indicates large lesions of the cell membrane allowing influx of PI and thus its death. Therefore, the increase of the PI fluorescence signal at the single-cell level most probably reflects the time-delayed influx of PI and its subsequent intercalation into the DNA. Interestingly, we could also observe cell lysis events that resulted in the release of red fluorescent nucleic acids (Figure 2b, (4)) which might also lead to subsequent decrease of cellular PI fluorescence signal intensities. Taken together, single-cell analysis of SOPP3-mediated phototoxicity clearly demonstrates a high, light-triggered antimicrobial efficacy against *E. coli* cells that is almost independent of the local environment at least in the used experimental setup. These properties make SOPP3 and the other genetically encoded photosensitizers suitable candidates for an application as light-driven antimicrobial agents in aPDI to fight human pathogens. However, for therapeutic applications PSs are usually added exogenously to the pathogenic organism instead of being synthetized intracellularly. For this reason, we next analyzed the extracellular phototoxicity of selected LOV-based and GFP-related PSs. 

### 2.2. Extracellular Phototoxicity of Different LOV- and GFP-Photosensitizer Proteins

**Figure 3:**
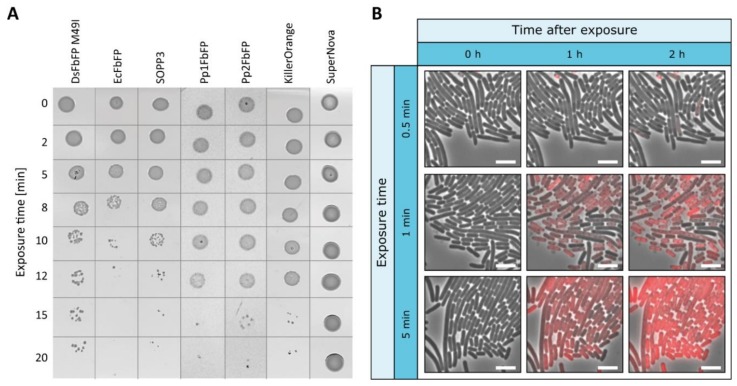
Extracellular antimicrobial activity of genetically encoded PSs on *E. coli* cells. (**a**) To investigate the effect of extracellularly added PSs, purified proteins have been analyzed by a plate spot assay. For this, *E. coli* cells were supplemented with the respective PS variants adjusted to an OD_450 nm_ (or OD_580 nm_ in the case of SuperNova) of 0.2 and then illuminated for different time periods with intense (~130 mW cm^−2^) blue (λ_max_ = 448 nm) or orange light (λ_max_ = 600 nm). Subsequently, 3 µL of the irradiated cells were dropped on agar plates and incubated at 37 °C overnight. To exclude blue light toxicity, a plate spot assay without the addition of a photosensitizer was performed as a control experiment (Supplementary Figure S3a). (**b**) Extracellular phototoxicity of SOPP3 on *E. coli* cells. Representative images from microfluidic experiments with extracellular SOPP3 (OD_450 nm_ = 0.3) addition and blue light exposure (λ_max_ = 445 nm). Images are shown for selected exposure times (0.5 min, 1 min, 5 min) and times after exposure (0 h, 1 h, 2 h). Scale bar = 5 µm.

To study the applicability of genetically encoded photosensitizers as light-activated antimicrobial agents, the phototoxicity of exogenous GFP-related and LOV-based PSs was further analyzed. In addition to SOPP3, SuperNova, and KillerOrange, different LOV-based PSs were used in this study exhibiting divergent ratios of type-I and -II photosensitizing activities and, hence, intracellular phototoxicities: besides EcFbFP and DsFbFP M49I, two LOV variants showing both type-I and type-II photosensitizing activities accompanied by comparatively high intracellular phototoxicities, Pp1FbFP and Pp2FbFP were chosen as PS derivatives with a moderate antimicrobial efficacy but selective singlet oxygen formation [47]. The extracellular killing efficiency of purified photosensitizing proteins has first been investigated in a plate spot assay using *E. coli* cells as a proof of concept. As shown in Figure 3a, exogenous addition of all tested PSs, except SuperNova, clearly affected the cell viability of *E. coli* cells after high intensity light irradiation (130 mW cm^−2^). However, in contrast to intracellularly located PSs, the exogenous approach required a longer exposure time of 8 (EcFbFP) to 15 min (Pp1-, Pp2FbFP, KillerOrange). In a control experiment, where *E. coli* cells were illuminated with possibly toxic blue light without adding purified PSs, no changes in growth behavior could be observed (Supplementary Figure S3a). 

To further analyze the accessibility of single *E. coli* cells to PS proteins that are assembled within a microcolony, the bacteria were cultivated in a microfluidic monolayer chip while adding purified SOPP3 to the growth medium (Figure 3b). As expected, illumination of extracellular PSs also resulted in almost complete cell death as indicated by increasing PI fluorescence in single cells. Remarkably, a similar heterogeneous PI pattern could be observed at an intermediate exposure time of 1 min, which is again independent of the relative position of bacterial cells within the microcolony. An irradiation of five minutes, however, also resulted in a strong PI signal that occurs homogenously within the whole *E. coli* population. Again, compared to the above described microfluidic observation with intracellularly expressed PSs, approximately 10-fold longer illumination times are necessary to reach a comparable phototoxic effect. Consequently, we could demonstrate that *E. coli* can efficiently and homogenously be killed using exogenously applied PS proteins.

**Figure 4:**
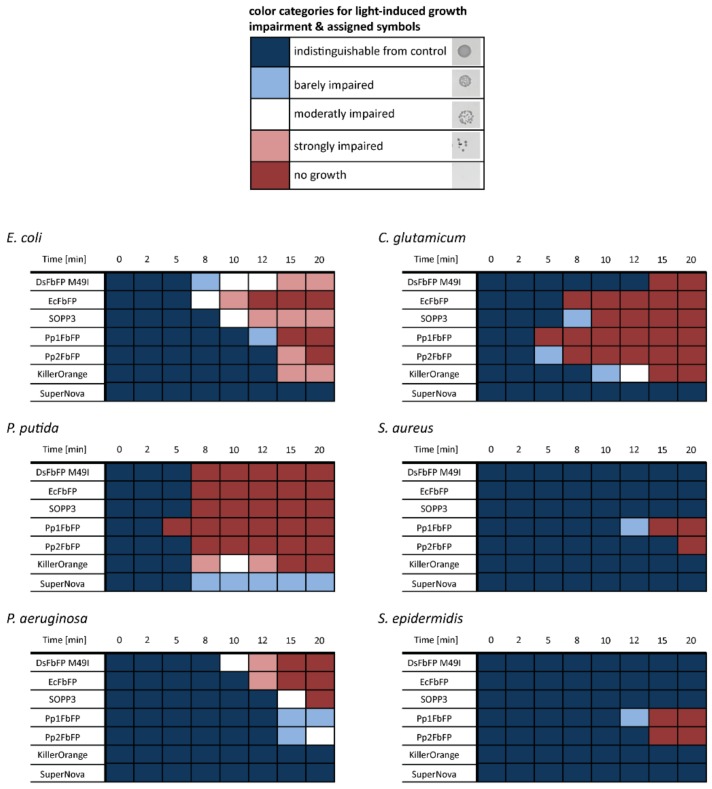
Extracellular antimicrobial activity of seven genetically encoded PSs against different bacterial strains. The growth responses of *E. coli*, *P. putida*, *P. aeruginosa*, *C. glutamicum*, *S. aureus,* and *S. epidermidis* were analyzed after addition of PSs by a plate spot assay. The detected colony formations were classified into five categories according to their cell survival: no growth impairment (dark blue), barely impaired growth (light blue), moderately impaired growth (white), strongly impaired growth (light red), and completely killed cells (dark red). For a better understanding of the data, the observed colony appearances, which correspond to the respective color categories, are shown in the upper panel.

Next, we tested the extracellular PS against the facultative pathogenic Gram-positive bacteria *Staphylococcus epidermidis* and *Staphylococcus aureus* [49,50] as well as *Corynebacterium glutamicum*, which is related to clinically concerning *Corynebacterium diphtheria* and *Mycobacterium tuberculosis* [51,52]. In addition, the Gram-negative non-pathogenic bacterium *Pseudomonas putida* and the opportunistic human pathogen *Pseudomonas aeruginosa*, whose carbapenem-resistant strains where grouped by the World Health Organization (WHO) into the “Priority 1: CRITICAL” class of pathogens [53], were included in the phototoxicity assay. All results of the plate spot assays are shown in Supplementary Figure S3b–f. Based on this data set and experimental setup, the phototoxic effect depended on the used photosensitizer, as well as the tested microorganisms, and light-induces growth impairment could roughly be classified into five categories as summarized in Figure 4. For example, *P. putida* is rather sensitive to extracellularly added photosensitizers, as illumination resulted in a pronounced growth impairment irrespective of the tested PS variants. Similar results were observed with *C. glutamicum* and *E. coli* where only SuperNova did not show a phototoxic effect. Remarkably, the pathogenic species exhibited an increased tolerance towards photodynamic inactivation. For instance, cell growth of *P. aeruginosa* was only significantly affected by DsFbFP M49I, EcFbFP, and SOPP3 (Figure 4 and Supplementary Figure S3f). In contrast, the Gram-positive pathogens *S. epidermidis* and *S. aureus* could only be killed by the variants Pp1- and Pp2FbFP that are known to be less-toxic while exclusively forming ^1^O_2_ via type-II reaction (Figure 4 and Supplementary Figure S3b,c [47]). This observation is in good agreement with previous reports describing that Gram-negative bacteria are more susceptible to O_2_^•−^, H_2_O_2_, and HO^•^ whereas Gram-positive bacteria show higher sensitivities towards ^1^O_2_ [54,55]. These differences might be explained by the divergent morphology: while Gram-negative bacteria possess an outer membrane that acts as an additional permeability barrier for extracellularly generated singlet oxygen, Gram-positive bacteria allow a direct translocation of the PS through the more permeable cell wall thereby facilitating a direct accessibility of the cytoplasmic membrane to this highly toxic ROS [56,57]. 

In this context, it should be noted that Westberg and coworkers could demonstrate by spectroscopic characterization that SOPP3 produces singlet oxygen with a quantum yield which fits to the quantum yield of FMN triplet state formation thereby implicating elimination of a competing type-I reaction in this engineered PS [41]. In addition, SOPP3 exhibits a very high singlet oxygen quantum yield (Φ_Δ_) of about 0.6 [41], as compared to Pp1- und Pp2-FbFP (Φ_Δ_ = 0.23 and 0.11) [47]. Therefore, we expected comparable antibiotic spectra of SOPP3, Pp1FbFP and Pp2FbFP for the tested bacteria but with differential phototoxicities (SOPP3 > Pp1FbFP > Pp2FbFP). However, the results presented here surprisingly indicate that the antimicrobial spectrum of SOPP3 rather resembles that of EcFbFP and DsFbFP M49I although these PSs are capable of efficiently producing O_2_^•−^ and H_2_O_2_ via type-I in addition to ^1^O_2_ producing type-II reaction [47]. To analyze, if differences in the antimicrobial spectra could be explained by the PS’s individual capabilities to perform type-I photosensitizing reactions under the tested exogenous conditions, we subsequently quantified O_2_^•−^ and H_2_O_2_ formation in vitro as described recently [47]. Therefore, the Amplex^®^Red reagent was used, which is converted to the red-colored product resorufin in the presence of H_2_O_2_. The reaction is catalyzed by a horseradish peroxidase (HRP) in a 1:1 stoichiometry, thus allowing a direct correlation between the detected absorption of resorufin and the generated hydrogen peroxide. Since the Amplex^®^Red assay detects hydrogen peroxide, a superoxide dismutase (SOD) was added to the reaction mixture in order to enzymatically convert O_2_^•−^ generated by the purified PS proteins upon illumination into H_2_O_2_. As expected, light exposure of EcFbFP and DsFbFP M49I resulted in considerable amounts of H_2_O_2_ formation after an illumination time of 5 min, whereas barely increased resorufin absorption could be detected in the samples containing Pp1- and Pp2FbFP thereby corroborating our previously published results (Supplementary Figure S4). Unexpectedly, KillerOrange and SuperNova showed a comparatively low type-I photosensitizing activity. In accordance with this observation, the two GFP-like PSs needed a much longer illumination time of up to 30 min to produce detectable amounts of H_2_O_2_, which could also explain the weak phototoxicities that were observed in the in vivo experiments. Surprisingly, SOPP3 exhibited the by far highest H_2_O_2_ production level, which is almost four times higher than EcFbFP and DsFbFP M49I. These results give thus a first indication that the specificity and efficiency of type-I and –II ROS formation seem to shape the efficacy and antimicrobial spectrum of the tested genetically encoded photosensitizers. However, especially the selectivity of ROS formation, inside and outside of living cells, clearly requires further examination by using appropriate detection methods under conditions relevant for the specific application.

**Figure 5:**
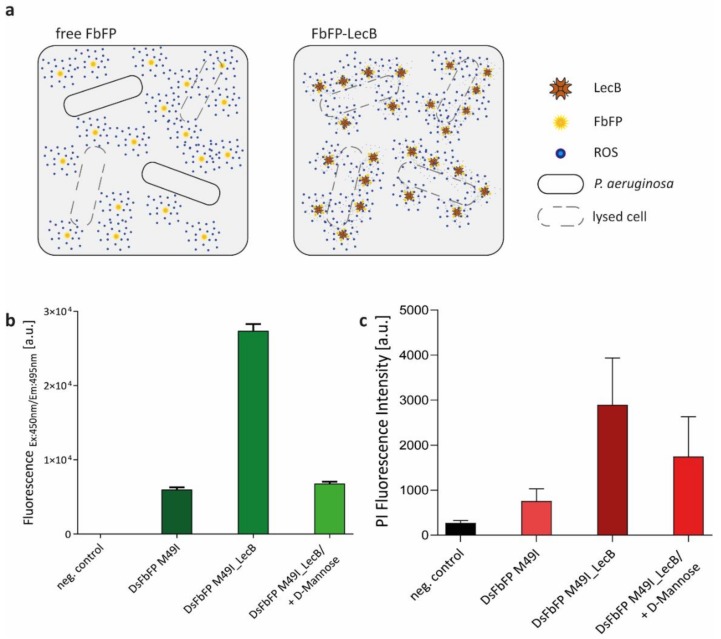
Phototoxicity towards *Pseudomonas aeruginosa* of a genetically encoded photosensitizer fused to the lectin LecB. (**a**) General strategy for improving extracellular phototoxicity of genetically encoded PSs by directing them to specific cellular structures. In this case, fusion with the lectin LecB can help to direct the PS to the *P. aeruginosa* cell envelope thereby allowing efficient killing of the pathogenic bacterium by light-driven ROS formation. (**b**) In order to test improved binding of DsFbFP M49I_LecB to *P. aeruginosa* cells, planktonic cells were mixed with DsFbFP or DsFbFP M49I-LecB (adjusted to a final OD_450 nm_ of 0.2), washed and subsequently investigated for FbFP fluorescence. In addition, the fusion protein DsFbFP M49I-LecB was incubated with d-mannose (final concentration 200 mg mL^−1^), which reduces fluorescence by targeted detachment from the cell surface. As a control experiment, *P. aeruginosa* cells without the addition of photosensitizers were measured. The data represent the mean values of three independent experiments and the error bars indicate the calculated standard deviations. (**c**) In vivo phototoxicity analysis was performed to evaluate the effect of DsFbFP M49I-LecB. To avoid that direct contact of PI and ROS, produced by extracellular PS upon illumination, leads to a decrease of PI fluorescence (see supplementary Figure S5 for details), the PI assay for monitoring cell viability was adapted by incubating the PSs with the *P. aeruginosa* cells first and then adding PI after a washing step. Both free and fused photosensitizers were used for comparison. As a control experiment, cells without the addition of DsFbFP M49I or DsFbFP M49I-LecB were used. The data represent the mean values of three independent experiments and the calculated standard deviations are shown.

Our data illustrated, as expected, that the localization of the applied PSs in either the intra- or extracellular space of bacterial cells plays an important role for the phototoxicity. Presumably, this is caused by variations of the PS concentration within the respective compartment, which, in turn, can directly influence the frequency of interactions between ROS and the ROS-sensitive cellular structures. To evaluate, if the extracellular phototoxicity of a protein-encased PS can be enhanced by specifically directing it to the bacterial cell envelope, we fused DsFbFP M49I, which exhibits the highest extracellular phototoxicity for the human pathogen *P. aeruginosa,* to the lectin LecB (Figure 5a). LecB is a multivalent sugar-binding protein derived from *P. aeruginosa* that is naturally formed by the bacterium for biofilm formation and initiation of human infections [58,59,60]. This lectin can bind to various sugar moieties located on the surface of *P. aeruginosa* cells [61,62] and it was recently published that LecB immobilized on the surface of hydrogels can be used to efficiently capture *P. aeruginosa* cells for their treatment with antimicrobial peptides [63]. Based on these findings, we analyzed if LecB can be used to facilitate the extracellular attachment of C-terminally fused DsFbFP M49I to *P. aeruginosa* for their efficient photodynamic inactivation. To analyze, if extracellular DsFbFP M49I-LecB shows improved cellular binding and phototoxicity, PS and PI fluorescence signals of washed cells were monitored in comparison to free DsFbFP M49I in the absence and presence of LecB-inhibiting d-mannose. The results shown in Figure 5b indicate that fusion with LecB enables DsFbFP M49I to bind over four times more efficiently to *P. aeruginosa* cells in the absence of d-mannose. More importantly, the extracellular antimicrobial phototoxicity of DsFbFP M49I-LecB increased 3.8-fold in comparison to the unmodified PS (Figure 5c). Consequently, the DsFbFP M49I-LecB fusion protein allowed an improved targeting and photodynamic inactivation of *P. aeruginosa*, presumably because more of the PS proteins are positioned close to the cell surface and ROS thus have to overcome shorter distances in order to damage cellular components.

## 3. Materials and Methods 

### 3.1. Construction of Expression Vectors

The genes of SOPP3, KillerOrange, and SuperNova were codon optimized for expression in *Escherichia coli*, *Rhodobacter capsulatus*, and *Pseudomonas putida* and obtained by commercial gene synthesis (Eurofins Genomics, Ebersberg, Germany; Geneart Gene Synthesis, distributed by Thermo Fisher Scientific, Regensburg, Germany) (Supplementary Figure S6). The genes were hydrolyzed with *Nde*I and *Xho*I whose restriction sites were placed during synthesis at 5′ and 3′ ends of each gene, respectively, and they were subsequently cloned into the *Nde*I and *Xho*I sites of the commercially available pET28a(+) vector (Novagen, distributed by Merck KGaA, Darmstadt, Germany). For generating the PS-LecB fusion protein, cloning was performed via the InFusion^®^ HD Cloning Plus kit (Takara Bio Europe, St Germain en Laye, France) as indicated by the supplier. Therefore, the DsFbFP M49I gene was amplified by PCR using primer pair 1/2 (Supplementary Table S1) (containing homologous sequences suitable for integration into the *Nho*I/*Xho*I hydrolyzed plasmid pURE [62], which in turn contains the *lecB* gene), and the plasmid pET28a-DsFbFP M49I as a template [47]. For the following purification a 6x-Histidine-tag was added to the previously constructed plasmid by mutagenesis primer pair 3/4 (Supplementary Table S1) (containing a His_6_-tag) and using the InFusion^®^ HD Cloning Plus kit (Takara Bio, Mountain View, CA, USA). All cloning experiments were conducted using *Escherichia coli* DH5α [64]. For isolation and purification of plasmid DNA from bacterial cells, the “innuPREP Plasmid Mini Kit” (Analytik Jena, Jena, Germany) was used. All newly constructed expression vectors were verified by DNA-sequencing (Eurofins Genomics, Ebersberg, Germany) (Supplementary Table S1).

### 3.2. Heterologous Expression and Purification

Expression of the genetically encoded PSs as well as the fusion constructs was performed in *E. coli* strain BL21(DE3) (Novagen #69450, distributed by Merck KGaA, Darmstadt, Germany). Cells were grown in 1 L auto-induction (AI) medium containing 47.6 g L^−1^ terrific broth (TB) (ready-to-use-mixture; Carl Roth, Karlsruhe, Germany), 0.05% glucose and 0.2% lactose in 5 L shake flasks at 37 °C for 24 h. For stable plasmid replication, media were supplemented with 50 µg mL^−1^ kanamycin or 100 µg mL^−1^ ampicillin, respectively. After harvesting, bacterial cells were resuspended in lysis buffer (50 mM NaH_2_PO_4_, 300 mM NaCl, pH 8.0) and disrupted using a French Press Cell Disrupter (Thermo Electron Corporation, Waltham, MA, USA). Supernatant of cell lysates was clarified by centrifugation (30 min, 4000 rpm, 4 °C) and subsequently applied to a 5 mL Ni^2+^-NTA metal-ion-exchange-chromatography-superflow-column (Qiagen, Hilden, Germany) with a flowrate of 2 mL min^−1^ and washed with eight column volumes of wash buffer (50 mM NaH_2_PO_4_, 300 mM NaCl, 50 mM imidazole, pH 8.0). Finally, the target proteins were eluted with the same buffer containing 250 mM imidazole. After purification, buffer exchange was performed in 10 kDa molecular-weight cutoff concentration units (Pall Corporation, New York, NY, USA). The sugar binding function and fluorescence activity of the DsFbFP M49I-LecB fusion partners were proven using a hemolysis assay and fluorescence spectrometry (Ackermann et al.). The purified proteins were stored in protein storage buffer (10 mM NaH_2_PO_4_, 10 mM NaCl, pH 8.0) at 4 °C in the dark.

### 3.3. Phototoxicity Analysis in Escherichia coli

To quantitatively determine the intracellular phototoxic effect of the photosensitizers, the colony forming capacity was measured in dependence on exposure time and light intensity. The experiment was performed as described before using light intensities of ~130 to 1 mW cm^−2^ and different illumination times (0 to 3 min) [47]. However, the illumination time was extended up to 30 min for the GFP-like photosensitizers as well as the empty vector control. Furthermore, for SuperNova, a light intensity of 85 mW cm^−2^ and 8 mW cm^−2^ at 580 nm was achieved by using a high-power LED (Nichia NCSA219B-V1 SMD-LED, amber, λ_max_ = 600 nm, 1 W; maximal light intensity at 600 nm = 138 mW cm^−2^, emission spectra are shown in supplementary Figure S7) placed at the top of a macro cuvette or upon 5.5 cm long spacers, respectively. The intensity was determined with the help of an optical power and energy meter (PM100D, Thorlabs, Newton, NJ, USA). Emission spectra of the used high-power LEDs were measured with a fluorescence spectrometer (Varian Cary Eclipse, Agilent Technologies, Ratingen, Germany). Additionally, the influence of a fourth light intensity (1 mW cm^−2^) was investigated. To reach the intensity of 1 mW cm^−2^ a programmable matrix of light-emitting diodes has been assembled with the assistance of Prof. Dr. Andreas Möglich as previously described [65]. For this approach, 1000 µL of the *E. coli* cells, which have been adjusted to a final OD_580 nm_ of 0.1 were transferred into a black colored 96-deepwell plate (Riplate^®^ RW, 43001-0216, black, Ritter GmbH, Schwabmünchen, Germany) and illuminated with the programmable LED matrix which has been directly placed on top of the deepwell plate.

Light-induced cell death was furthermore analyzed using propidium iodide (PI) which is a fluorescent dye that selectively enters dead cells and shows a significant fluorescence at λ = 617 nm after intercalation into DNA [66]. The experiment was performed with *E. coli* BL21(DE3) cells (Novagen, distributed by Merck KGaA, Darmstadt, Germany) transformed with recombinant pET28a(+) expression vectors (Supplementary Table S1) which allows the expression of the respective photosensitizers. The expression culture was inoculated to an OD_580 nm_ of 0.05 in Lysogeny Broth (LB) medium (Carl Roth, Karlsruhe, Germany) supplemented with 50 µg mL^−1^ kanamycin. Cultivation was performed in a 48-well microtiter plate (FlowerPlate, m2p-labs GmbH, Baesweiler, Germany) with a final volume of 1 mL at 37 °C and 1200 rpm. After induction with 0.4 mM IPTG at an OD_580 nm_ of 0.6 to 0.8, the cells were incubated for 3 h under continuous shaking. Subsequently, the cells were adjusted to an OD_580 nm_ of 0.5, washed with PBS buffer (pH 7.4) and finally resuspended in 1 mL PI assay buffer (100 µM EDTA, 5 µM PI in PBS, pH 7.4). The samples were transferred into another FlowerPlate and illuminated with blue light (λ = 447 nm, 10 mW cm^−2^) in a microbioreactor (BioLector, m2p-labs GmbH, Baesweiler, Germany). In the case of SuperNova, spacers of 5.5 cm were positioned between the sample and the high-power LED (Nichia NCSA219B-V1 SMD-LED, amber, λ = 600 nm, 1 W, resulting in a light intensity of 8 mW cm^−2^ at 580 nm) as described above. At several time points, 100 µL of the irradiated cell solution were transferred into a 96-well microtiter plate (Greiner Bio-One GmbH, Frickenhausen, Germany) and analyzed regarding the respective PS fluorescence (LOV-based PS: λ_ex_ = 450 nm, λ_em_ = 495 nm; KillerOrange: λ_ex_ = 450 nm, λ_em_ = 555 nm; SuperNova: λ_ex_ = 580 nm, λ_em_ = 610 nm) as wells as PI fluorescence (λ_ex_ = 535 nm, λ_em_ = 617 nm) using a microplate reader (Infinite^®^ M1000 Pro, Tecan Group LTD., Maennedorf, Switzerland). For evaluation, the PI fluorescence was normalized to the expression coefficient (ExCo) to determine PS-mediated phototoxicity regardless of its expression efficiency in *E. coli* [47]. The normalized PI intensity (I_n_) was determined using equation 1, where I_raw_ is the raw PI fluorescence, I_PS_ the LOV-FP-fluorescence intensity of the cell culture before blue light illumination, Φ_F_ the fluorescence quantum yield and ε the molar extinction coefficient of the respective LOV-FP (equation 1):

(1) $$I_{n}=\;\frac{I_{raw}}{\left(\frac{I_{PS}}{\Phi_{F}\;x\;\varepsilon }\right)}$$

The following values were used for calculation of the normalized PI intensity (I_n_): EcFbFP (ε = 14,500 M^−1^ cm^−1^; Φ_F_ = 0.44) [46], SOPP3 (ε = 15,000 M^−1^ cm ^−1^; Φ_F_ = 0.41) [41], KillerOrange (ε = 22,600 M^−1^ cm^−1^; Φ_F_ = 0.42) [26], SuperNova (ε = 33,600 M^−1^ cm^−1^; Φ_F_ = 0.30) [25].

### 3.4. Single-Cell Cultivation and Analysis

To address the question whether the PI signal and with that the light-induced damage of cells via ROS generation occurs homogenously within in the culture, single-cell analysis has been carried out using flow cytometry and microfluidic analysis.

#### 3.4.1. Flow Cytometry (FCM)

Flow cytometry that allows multiparametric analysis of cellular characteristic, has been used to analyze bacterial cells regarding their intrinsic fluorescence and cell viability using PI. Based on the PI assay, *E. coli* BL21(DE3) cells (Novagen, distributed by Merck KGaA, Darmstadt, Germany) harboring the expression vector pET28a-SOPP3 has been cultivated in LB medium (Carl Roth, Karlsruhe, Germany) supplemented with 50 µg mL^−1^ kanamycin and inoculated with an OD_580 nm_ of 0.05 in 100 mL shake flasks at 37 °C and 120 rpm. *E. coli* cells carrying the empty vector were used as an appropriate control experiment. When cultures reached an optical density of 0.6 to 0.8 (approximately after an incubation time of 2 h) gene expression was induced by adding 0.4 mM IPTG and cultivation was continued at the same conditions for 3 h. Then, the cells were harvested by centrifugation (2 min, 15,000 rpm, RT), washed in PBS buffer (pH 7.4) and finally adjusted to an OD_580 nm_ of 0.5 in 1 mL PI assay buffer containing PBS, 100 µM EDTA and 5 µM PI and then transferred into a 48-well microtiter plate (Round Well Plate, m2p-labs GmbH, Baesweiler, Germany). Illumination was performed using a BioLector (λ = 447 nm, 10 mW cm^−2^, m2p-labs GmbH, Baesweiler, Germany). After 0, 10 and 60 min, aliquots (100 µL) of the irradiated cultures were taken and analyzed with a flow cytometer (Amnis^®^ CellStreamTM System, Merck, now Luminex Corporation, Austin, USA). Samples were analyzed using a 561 nm-laser with a maximum power of 150 mW and fluorescence was detected by a 611/31 nm (red) bandpass filter. Furthermore, the intrinsic SOPP3 fluorescence was measured with a 488 nm-laser (maximal laser power of 200 mW) and detected by a 528/46 nm bandpass filter. To exclude cell debris and cell accumulations, the cells were also analyzed regarding their size (forward scatter, FSC) and granularity (side scatter, SSC). FSC was measured using a FSC laser with 30% of the laser power (456/51 nm bandpass filter) and for the SSC a dedicated laser with 100% of the laser power (773/56 nm bandpass filter) was used. Based on the scatter plots, bacterial cells were gated from irrelevant counts for fluorescence analysis. Flow cytometric data were evaluated with the CellStreamTM Analysis Software (Merck, now Luminex Corporation, Austin, TX, USA).

#### 3.4.2. Microfluidic Chip Design and Fabrication

Disposable polydimethylsiloxane (PDMS) chips for single-cell analysis were fabricated as previously described [67,68]. In short, photolithography was used for the production of a structured silicon wafer, which was used as a master mold for PDMS softlithography. The microfluidic chips consist of three arrays of cultivation chambers with 50 cultivation chambers each (dimensions of one cultivation chamber: 1 µm × 60 µm × 70 µm). The chamber height of 1 µm restricts cell growth to a monolayer, allowing the analysis of cell growth with full spatio-temporal resolution [69]. The chamber arrays are interconnected by parallel-arranged 10 µm deep supply channels.

#### 3.4.3. Microfluidic Single-Cell Cultivation

Prior chip inoculation cells were pre-cultured in 100 mL (filling volume: 10 mL) shake flasks until the OD_580 nm_ reached a value of 0.5. The chip was inoculated with cell suspension until a few cells got randomly trapped in the cultivation chambers [70]. Afterwards, the cells were continuously perfused with fresh medium using a flow rate of 200 nL min^−1^. During cultivation the chip was kept at 37 °C. All microfluidic experiments for the analysis of intracellular phototoxicity were performed with *E. coli* Tuner(DE3) harboring pET28a-SOPP3. After chip inoculation, cells were cultivated for 1.5 h with a continuous supply of LB medium containing additionally 0.1 mM IPTG, 0.1 mM EDTA, and 0.1 µM PI. Subsequently, single chambers were exposed to blue light (fluorescence filter λ_max_ = 445 nm) with exposure times ranging from 100 ms to 10 s. Extracellular phototoxicity of SOPP3 was tested with *E. coli* Tuner(DE3) wild-type cells that were grown in the microfluidic chip for 1.5 h with continuous LB medium supply. Then the medium was exchanged with LB medium containing SOPP3 (OD_450 nm_ = 0.3) and 0.1 µM PI. A higher medium flow (900 nL min^−1^) was applied to exchange the medium rapidly. After 20 min the flow was stopped to induce batch-like conditions [48] and single chambers were exposed to blue light with times ranging from 30 s to 5 min.

#### 3.4.4. Live-Cell Imaging and Image Analysis

Microfluidic experiments were performed on an inverted automated microscope (Nikon Eclipse Ti, Nikon, Tokyo, Japan), equipped with a laser assisted focus system for optimal imaging. A benchtop incubation chamber (PECON, Germany) ensured optimal temperature conditions. The inlets of the microfluidic systems were connected to a syringe pump system (neMESYS, CETONI, Korbussen, Germany) for continuous medium supply. Nikon software NIS Elements AR 4.30.02 was used for automated time-lapse imaging. The microfluidic chip was placed in an in-house fabricated chip-holder and phase contrast and fluorescence images were taken every 10 min using a 100x oil immersion objective (CFI Plan Apo Lambda DM 100×-magnification, NA 1.45).

The PI fluorescence was captured with a mCherry filter (λ_ex_ = 562 nm, λ_em_ = 641 nm, DM= 593 nm) and blue light was applied with a blue light filter (λ_ex_ = 445 nm, λ_em_ = 494 nm, DM = 470 nm). Fluorescence light intensity was set to 10% of maximum intensity (max. 3.5 W white light output).

Segmentation was performed using the neural network-based segmentation tool JUNN (Sachs et al.), which is based upon the U-Net [71] neural network structure. Segmented images were further processed with the open source software Fiji [72]. Phototoxicity was quantified by an increase in PI fluorescence inside the cells. The cell area for each image was calculated and mean fluorescence intensities of each chamber was determined by measuring the fluorescence value and subtracting the background fluorescence.

### 3.5. Extracellular Phototoxicity Analysis

Analysis of the extracellular phototoxicity was performed with six different bacterial strains, including Gram-negative and -positive organisms. Cultivation was performed in LB medium (Carl Roth, Karlsruhe, Germany) in 100 mL (filling volume: 10 mL) shake flasks inoculated with an OD_580 nm_ of 0.05 of the respective bacterial strain. Cultures containing cells of *E. coli* BL21(DE3), *Pseudomonas aeruginosa* PAO1 (ATCC: 27853), *Staphylococcus aureus* (ATCC: 25923) or *S. epidermidis* (ATCC: 12228) were incubated at 37 °C for 5 h. For cultivating *P. putida* KT2440 (ATCC: 47054) or *Corynebacterium glutamicum* (ATCC: 13032) cells, a cultivation temperature of 30 °C was set. After incubation, the cells were diluted to a cell density corresponding to an OD_580 nm_ of 0.25 in PBS buffer (pH 7.4). Subsequently, 15 µL of the cells were transferred into a macro cuvette, purified photosensitizer was added with a final absorption of 0.2 at its absorption maximum (450 nm or 580 nm) and the suspension was then adjusted to a final volume of 150 µL with 1x PBS buffer (pH 7.4). The macro cuvette was directly placed on top of a blue (Nichia NCSB219B-V1 SMD-LED, royal blue, λ_max_ = 448 nm, 130 mW cm^−2^) or an orange light-emitting LED (Nichia NCSA219B-V1 SMD-LED, amber, λ_max_ = 600 nm, 138 mW cm^−2^). In order to ensure a constant room temperature during exposure, cooling units were installed on both sides of the cuvette. At given time points (0 to 20 min), 3 µL aliquots were taken out of the irradiated cell solutions and dropped on LB agar plates. Additionally, light-induced effects in the absence of extracellularly supplied photosensitizers were analyzed for cultures of all bacterial strains. The agar plates were incubated at 37 °C or in case of *P. putida* KT2440 and *C. glutamicum* at 30 °C overnight. Phototoxicity of extracellularly added PSs is indicated by growth impairment.

To adapt the PI assay to the requirements of extracellular PS addition, some preliminary experiments with pure DNA were performed. 10 μL salmon sperm (1 mg mL^−1^) (Sigma-Aldrich Chemie GmbH, Hamburg, Germany) was used and EcFbFP was added according to an OD_450 nm_ of 0.2. The solution was then filled up to 90 μL with 1x PBS buffer. Depending on the test conditions, 10 μL PI buffer was added before or after exposure. The whole suspension was transferred into a 96-well microtiter plate (Greiner Bio-One GmbH, Frickenhausen, Germany), which was directly placed on top of a blue light-emitting LED (λ_max_ = 448 nm; 130 mW cm^−2^). The PI fluorescence signal (λ_Em_ = 535 nm; λ_Ex_ = 617 nm) was measured after an illumination time of 20 min with the microplate reader (Infinite^®^ M1000 Pro, Tecan Group LTD., Maennedorf, Switzerland).

For the DsFbFP M49I-LecB fusion protein, a modified PI assay was performed. After incubation, *P. aeruginosa* PAO1 cells were diluted to a cell density corresponding to an OD_580 nm_ of 0.25 in 1x PBS buffer (pH 7.4). Subsequently, 10 µL of culture suspension were transferred into a reaction tube and the purified fusion protein was added resulting in a final absorption (OD_450 nm_) of 0.2. As a control, 20 µL of a 1 g mL^−1^ stock solution d-Mannose was additionally added. The suspension was then adjusted to a final volume of 90 µL with 1x PBS buffer (pH 7.4). After an incubation time of 1 h, the whole suspension was transferred into a 96-well microtiter plate (Greiner Bio-One GmbH, Frickenhausen, Germany), which was directly placed on top of a blue light-emitting LED (λ_max_ = 448 nm; 130 mW cm^−2^). After an illumination time of 20 min, 10 µL PI buffer (10×) was added. The PI signal (λ_ex_ = 535 nm, λ_em_ = 617 nm) was then measured with a microplate reader (Infinite^®^ M1000 Pro, Tecan Group LTD., Maennedorf, Switzerland).

### 3.6. Statistical Analysis

Data management and analysis were carried out using Microsoft Excel 2010 (Microsoft Corporation, Redmond, WA, USA) and GraphPad Prism 8.0.2 (GraphPad Software, San Diego, CA, USA). The datasets generated during this work are available from the corresponding author on reasonable request.

## 4. Conclusions

Current approaches to improve photodynamic therapies include the development and application of genetically encoded photosensitizers. This relatively new class of PSs exhibits a robust photochemistry of the photosensitizing chromophore, which is less prone to influences of the surrounding environment. In addition, linked protein domains with defined binding specificities principally allow directing the phototoxic agent to ROS-sensitive cellular structures. Thus, topically or locally delivered PSs with genetically engineered binding specificity could help to selectively and non-invasively treat multi-drug resistant pathogens proliferating in wounds, burns, or soft tissues in the near future. In this context, our data give a first indication that the ROS type and yield as well as the localization of the applied PS protein can strongly influence the antibacterial spectrum and efficacy. To this end, specificity of PS-mediated ROS formation by type-I and -II reactions, as well as the contribution of different ROS to the antimicrobial efficacy against Gram-positive and -negative pathogens, opens up new opportunities for efficient treatment of various life-threatening pathogens. Clearly, however, there is a need for further investigation.

## Supplementary Materials

Supplementary materials can be found at https://www.mdpi.com/1422-0067/20/18/4608/s1.

## Abbreviations

aPDI	Antimicrobial photodynamic inactivation
PDT	Photodynamic therapy
PS	Photosensitizer
ROS	Reactive oxygen species
ISC	Intersystem crossing
FP	Fluorescent protein
GFP	Green fluorescent protein
FbFP	Flavin-binding protein
LOV	Light-oxygen-voltage
SOG	Singlet Oxygen Generator
CALI	Chromophore-assisted light inactivation
SOPP	Singlet oxygen photosensitizing protein
LED	Light-emitting diode
CFU	Colony forming units
PI	Propidium iodide
FCM	Flow cytometry
FSC	Forward scatter
SSC	Side scatter
WHO	World Health Organization
HRP	Horseradish peroxidase
SOD	Superoxide dismutase
AI	Auto-induction medium
TB	Terrific Broth medium
LB	Lysogeny Broth medium
NTA	Nitrilotriacetic acid
OD	Optical density
LB	Lysogeny Broth medium
PBS	Phosphate buffer saline
ExCo	Extinction coefficient
PDMS	Polydimethylsiloxane
DM	Dichroic mirror
IPTG	Isopropyl-β-d-1-thiogalactopyranoside
