# Supplementary material for: Genetically Encoded Photosensitizers as Light-Triggered Antimicrobial Agents
Source: Int J Mol Sci. 2019 Sep 17;20(18):4608. doi: 10.3390/ijms20184608 (PMC6769541; doi:10.3390/ijms20184608)
Supplement: Supplementary file 1 [file ijms-20-04608-s001.pdf]

Supplementary Figures

Genetically Encoded Photosensitizers as Light-Triggered Antimicrobial Agents

Fabienne Hilgers [1,†], Nora Lisa Bitzenhofer [1,†], Yannic Ackermann [1], Alina Burmeister [2,3], Alexander Grünberger [2,3], Karl-Erich Jaeger [1,3] and Thomas Drepper [1,*]

[1] Institute of Molecular Enzyme Technology, Heinrich-Heine-University Düsseldorf, Forschungszentrum Jülich GmbH, D-52428 Jülich, Germany
[2] Multiscale Bioengineering, Bielefeld University, D-33501 Bielefeld, Germany
[3] Institute of Bio- and Geosciences, IBG-1: Biotechnology, Forschungszentrum Jülich GmbH, D-52428 Jülich, Germany
† These authors contributed equally to this work.
* Correspondence: t.drepper@fz-juelich.de

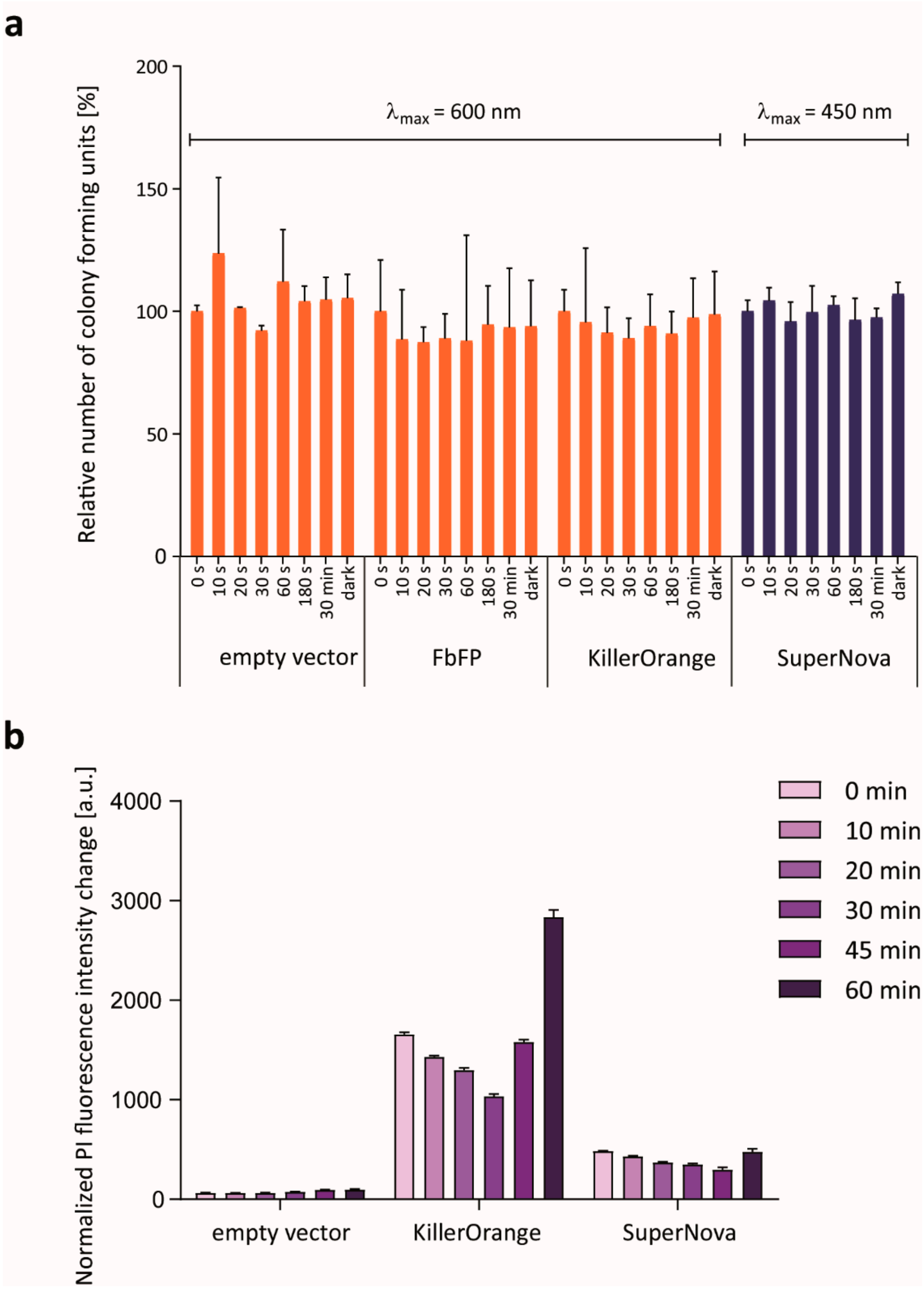

Figure S1: Control experiment for the evaluation of the wavelength- and light intensity-dependent phototoxicity of the individual PSs. (**a**) Evaluation of the wavelength-dependent photoactivation of the individual PSs using colony forming units (CFU). Control experiment for the evaluation of the wavelength-dependent photoactivation of the individual PSs using colony forming units (CFU). To exclude an influence on orange light ($\lambda_{,max}$ = 600 nm) on the LOV-based PSs, KillerOrange and the empty vector control as well as an influence of blue light (λ_{max} = 448 nm) on SuperNova, *E. coli* BL21 (DE3) harboring the respective expression vectors was used. After cultivation of expression cultures, cells were diluted to a finale $OD_{580\ nm}$ of 0.1 in PBS buffer (pH 7.4) and subsequently illuminated with the according light source (~130 mW cm^{-2}) for different illumination times. Aliquots of the irradiated samples have been transferred to LB agar plates and incubated over night at 37 °C in the dark. Data represent the mean values of the CFUs from three independent measurements. The corresponding standard deviations are indicated by error bars. (**b**) Quantitative in vivo phototoxicity studies of KillerOrange and SuperNova at high light intensities using the propidium iodide (PI) cell death assay. For the PI-based cell death assay, *E. coli* cells producing the PS KillerOrange and SuperNova were

adjusted to an $OD_{580 nm}$ of 0.5 in PI assay buffer (pH 7.4) and illuminated with high light intensities of blue (130 mW cm^{-2}, λ_{max} = 447 nm) or orange light (138 mW cm^{-2}, λ_{max} = 600 nm). The bars indicate the change in PI fluorescence intensity (λ_{ex} = 535 nm; λ_{em} = 617 nm) in dependence on the exposure time. The data were normalized to the amount of functional protein per cell, to exclude an influence of different protein accumulation levels. The data represent the mean values of three independent experiments and the error bars indicate the calculated standard deviations.

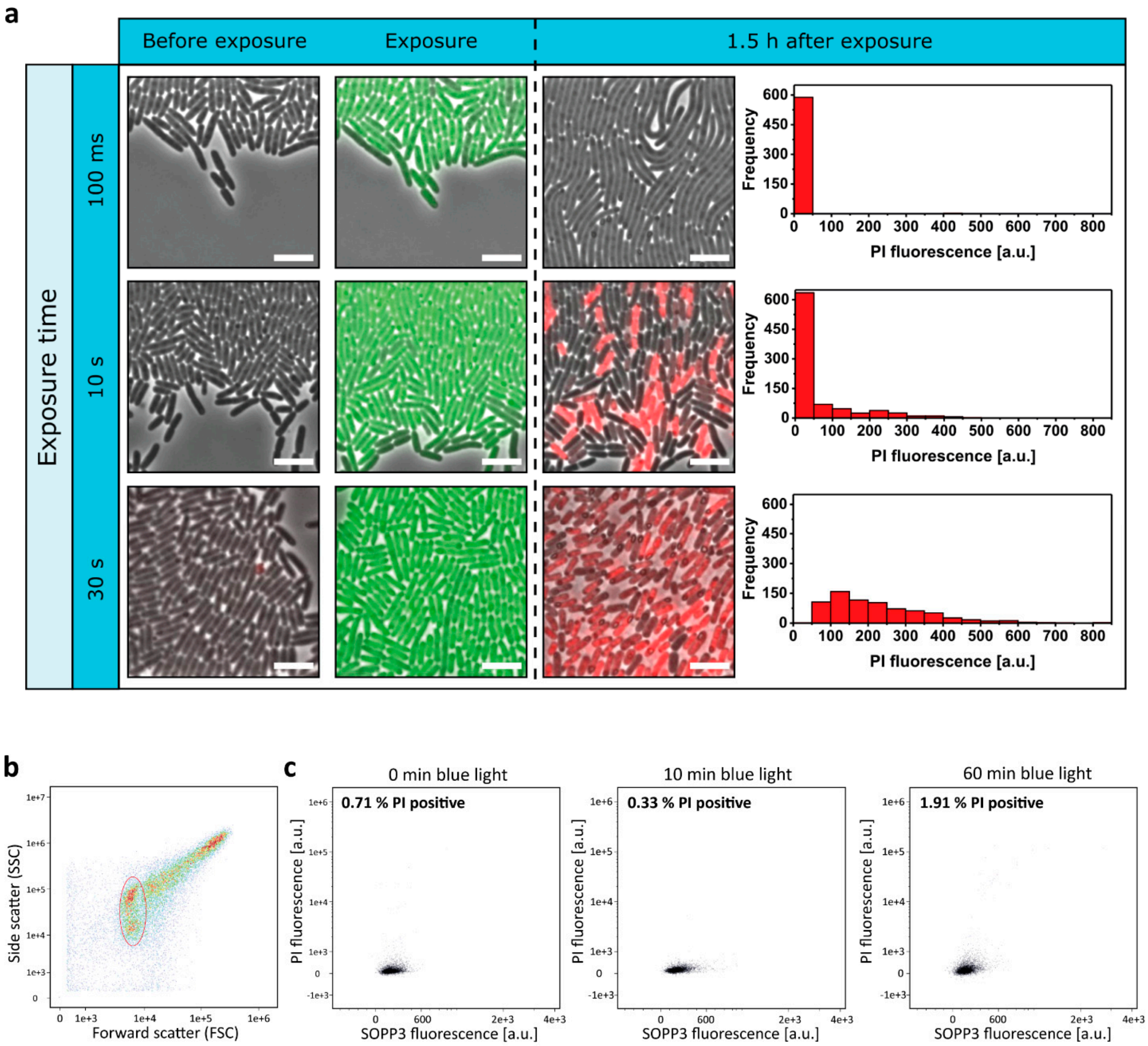

Figure S2: In vivo phototoxicity studies using the propidium iodide (PI) cell death assay as a quantitative marker for dead cells. (**a**) Images from microfluidic experiments with intracellular SOPP3 expression and different blue light exposure times. Images are shown for selected exposure times (100 ms, 10 s, 30 s) and time points during cultivation (before blue light exposure, at blue light exposure, 1.5 h after exposure). Both, the homogeneous distribution of SOPP3 expressed in individual cells of the microcolony at the time of exposure (indicated by the green fluorescence) and the PI fluorescence distributions 1.5 h after exposure for the corresponding exposure times are shown. Dead cells were stained red. The respective graphs represent the frequency of different PI fluorescence intensities within one microfluidic cell 1.5 h after exposure. While an exposure of 100 ms did not lead to PI positive cells at all and 10 s of blue light only addressed a few cells, the exposure of 30 s showed a nearly homogeneous PI fluorescence signal within the whole chamber. Scale bar = 5 µm. (**b)** Scatter plot of side versus forward scatter of *E. coli* cells harboring the expression vector pET28a-SOPP3, to identify cells of interest. A density plot was used to display the scattering. Regions with many events are displayed in red, regions with moderate events in green and regions with few events in blue. The gated population is circled in red. Doublets and cell accumulations were excluded with the help of the event gallery. This is a device-specific camera-enabled feature of the CellStream acquisition software, which displays a live flow of the sample in the channels to allow population verification and discrimination of duplicates. (**c**) *E. coli* BL21(DE3) cells harboring an empty vector were analyzed for fluorescence analysis and gated based on FSC and SSC to exclude cell debris and accumulation of cells. The fluorescence intensity of propidium iodide was measured using a 561 nm-laser (and a 611/31 nm (red) bandpass filter) and plotted using a log scale. Additionally, the intrinsic fluorescence was

analyzed with a 488 nm-laser and detected by a 528/46 nm bandpass filter. Dead *E. coli* cells (presented as red populations) are shifted to higher log values of the axis of abscissas and the percentage of dead cells is displayed in the upper left corner. Living cells are represented as black populations.

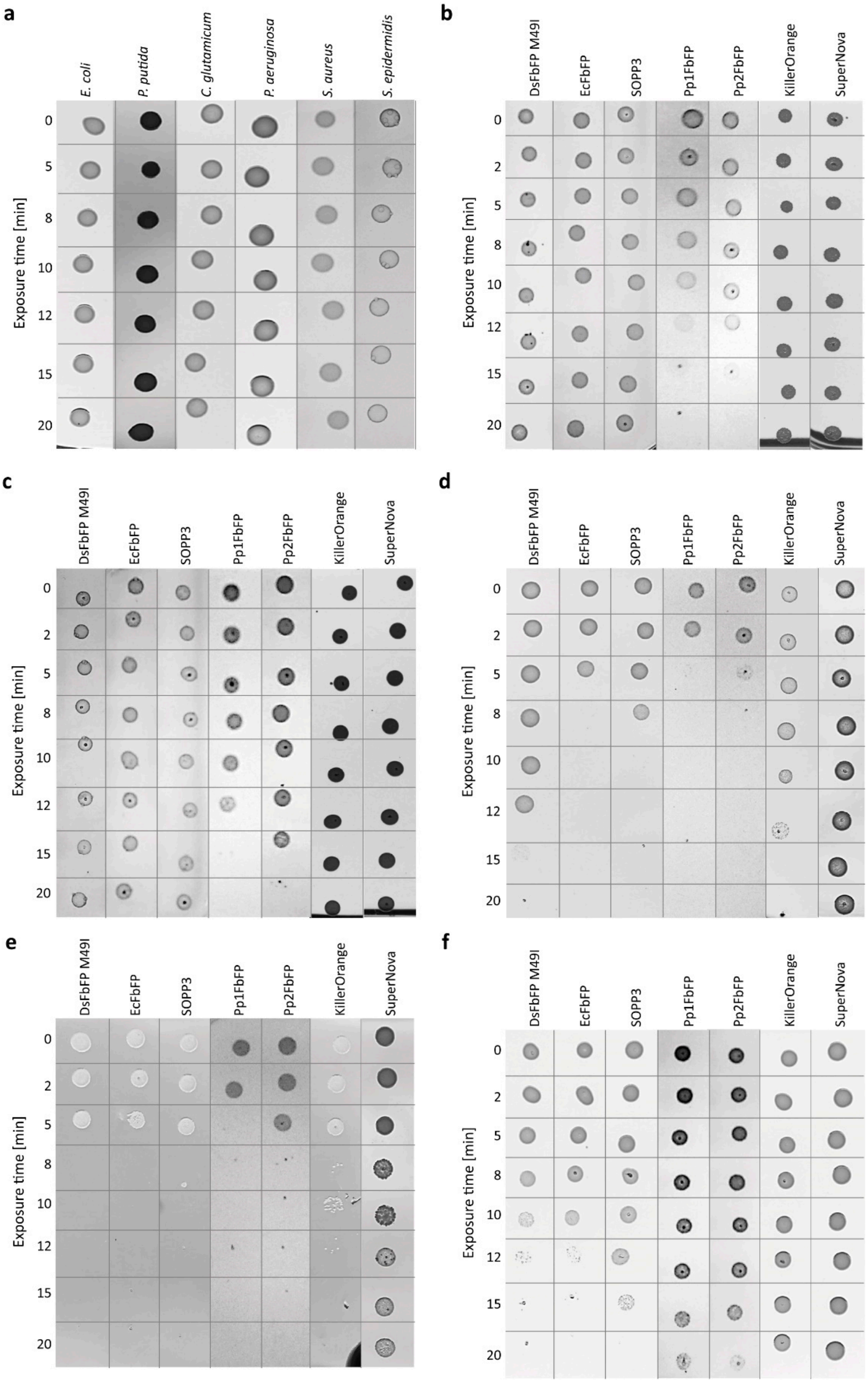

Figure S3: Extracellular antimicrobial activity of genetically-encoded PSs on bacteria. To investigate the effect of extracellularly added PSs, purified proteins have been analyzed by a plate spot assay. For this, bacterial cells ((**b**) *S. epidermidis* 12228; (**c**) *S. aureus* 25923; (**d**) *C. glutamicum* 13032; (**e**) *P. putida* KT2440; (**f**) *P. aeruginosa* PAO1) have been supplemented with the respective PS variant and then illuminated for different time periods with intense blue (λ_{max} = 448 nm, 130 mW cm^{-2}) or orange light (λ_{max} = 600 nm, 130 mW cm^{-2}). Subsequently, 3 μL of the irradiated cells were dropped on agar plates and incubated overnight. To exclude blue light toxicity, a plate spot assay without the addition of a photosensitizer has been performed as a control experiment (**a**).

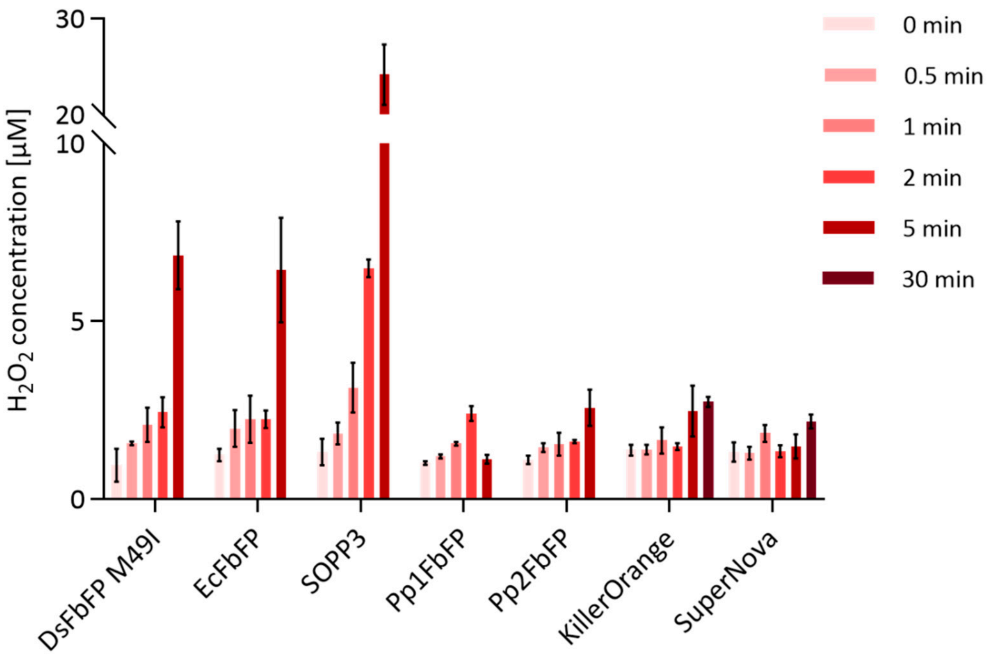

Figure S4: Quantification of PS-catalyzed hydrogen peroxide formation. Determination of H_2O_2, generated by DsFbFP M49I, EcFbFP, SOPP3, Pp1FbFP, Pp2FbFP, KillerOrange and SuperNova, was performed with the Amplex®Red Hydrogen Peroxide/Peroxidase Assay Kit (Molecular Probes, Invitrogen, part of Thermo Fisher Scientific, Eugene, USA) using purified photosensitizers adjusted to an $OD_{450\ nm}$ (LOV-based PSs and KillerOrange) or $OD_{580\ nm}$ (SuperNova) of 0.05. The measurements were performed according to the manufacturer's manual and as described by Endres *et al.* (2018) [1]. To accurately determine differences in ROS formation, the PSs have been illuminated with low light intensities (~10 mW cm^{-2}) with blue (λ_{max} = 447 nm) or orange (λ_{max} = 600 nm) light before adding the Amplex®Red reagent. Resorufin production (the product of the Amplex®Red reaction) was photometrically measured at 560 nm. To determine the final H_2O_2 concentration, a calibration curve was prepared (0 μM to 50 μM). The data represent the values of three independent experiments and the calculated standard deviations are indicated by error bars.

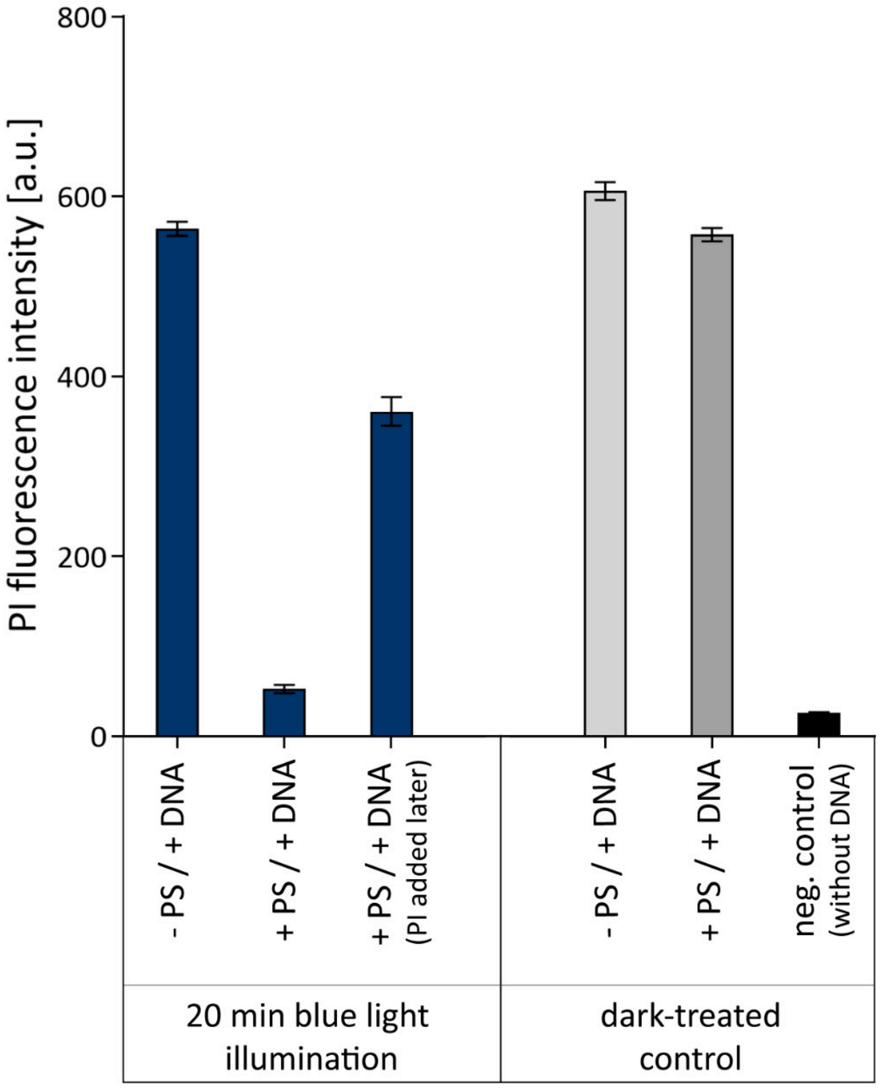

Figure S5: In vitro analysis to determine the influence of ROS on propidium iodide (PI). An in vitro experiment was performed to investigate the effect of PS-formed ROS on PI. The photosensitizer EcFbFP was mixed with salmon sperm DNA in PI assay buffer with or without PI and exposed to blue light (λ_{max} = 448 nm; 130 mW cm^{-2}) for 20 min. The PI fluorescence was then measured at λ_{ex} = 535 nm and λ_{em} = 617 nm. Samples without PI addition were supplemented with PI after illumination and prior to fluorescence detection. Unexposed samples as well as a negative control without the addition of DNA were carried as controls. The data represent the mean values of three independent experiments and the error bars indicated the calculated standard deviations.

Codon optimized DNA sequences of photosensitizing proteins for expression in *E. coli*, *P. putida* and *R. capsulatus* [a].

(a) SOPP3 (codon optimized) from *Arabidopsis thaliana* [2]

GGATCCATGGCATATGGAAAAAAGCTTTGTGATTACCGATCCGCGCCTGCCGGATAACCCGATTATTTTT
GCGAGCGATGGCTTTCTGGAACTGACCGAATATAGCCGCGAAGAAATTCTGGGCCGCAACGGCCGCTTT
CTGCAGGGCCCGGAAACCGATCAGGCGACCGTGCAGAAAATTCGCGATGCGATTCGCGATCAGCGCGA
AATTACCGTGCAGCTGATTAACTATACCAAAAGCGGCAAAAAATTTCTGAACCTGCTGAACCTGCAGCC
GATTCGCGATCAGAAAGGCGAACTGCAGGCGTTTATTGGCGTGGTGCTGGATGGCTAAGAATTCCTCGAG

(b) SuperNova (codon optimized) from *Anthomedusae sp* [3]
GGATCCATGGCATATGGGCAGCGAAGTGGGCCCGGCGCTGTTCCAGAGCGATATGACCTTCAAAATCTT
CATCGATGGCGAAGTGAACGGCCAGAAATTCACCATCGTGGCGGATGGCAGCAGCAAATTCCCGCATGG
CGATTTCAACGTGCATGCGGTGTGCGAAACCGGCAAACTGCCGATGAGCTGGAAACCGATCTGCCATCT
GATCCAGTATGGCGAACCGTTCTTCGCGCGCTATCCGGATGGCATCAGCCATTTCGCGCAGGAATGCTTC
CCGGAAGGCCTGAGCATCGATCGCACCGTGCGCTTCGAAAACGATGGCACCATGACCAGCCATCATACC
TATGAACTGGATGATACCTGCGTGGTGAGCCGCATCACCGTGAACTGCGATGGCTTCCAGCCGGATGGCC
CGATCATGCGCGATCAGCTGGTGGATATCCTGCCGAGCGAAACCCACATGTTCCCGCATGGCCCGAACG
CGGTGCGCCAGACCGCGACCATCGGCTTCACCACCGCGGATGGCGGCAAAATGATGGGCCATTTCGATA
GCAAAATGACCTTCAACGGCAGCCGCGCGATCGAAATCCCGGGCCCGCATTTCGTGACCATCATCACCA
AACAGACCCGCGATACCAGCGATAAACGCGATCATGTGTGCCAGCGCGAAGTGGCGTATGCGCATAGC
GTGCCGCGCATCACCAGCGCGATCGGCAGCGATGAAGATTGAGAATTCCTCGAG

(c) KillerOrange (codon optimized) from *Anthomedusae sp* [4]
GGATCCATGGCATATGGAATGCGGCCCGGCGCTGTTCCAGAGCGATATGACCTTCAAAATCTTCATCGAT
GGCGAAGTGAACGGCCAGAAATTCACCATCGTGGCGGATGGCAGCAGCAAATTCCCGCATGGCGATTTC
AACGTGCATGCGGTGTGCGAAACCGGCAAACTGCCGATGAGCTGGAAACCGATCTGCCATCTGATCCAG
TGGGGCGAACCGTTCTTCGCGCGCTATCCGGATGGCATCAGCCATTTCGCGCAGGAATGCTTCCCGGAAG
GCCTGAGCATCGATCGCACCGTGCGCTTCGAAAACGATGGCACCATGACCAGCCATCATACCTATGAAC
TGAGCGATACCTGCGTGGTGAGCCGCATCACCGTGAACTGCGATGGCTTCCAGCCGGATGGCCCGATCAT
GCGCGATCAGCTGGTGGATATCCTGCCGAGCGAAACCCACATGTTCCCGCATGGCCCGAACGCGGTGCG
CCAGCTGGCGTTCATCGGCTTCACCACCGCGGATGGCGGCCTGATGATGGGCCATCTGGATAGCAAAAT
GACCTTCAACGGCAGCCGCGCGATCGAAATCCCGGGCCCGCATTTCGTGACCATCATCACCAAACAGAT
GCGCGATACCAGCGATAAACGCGATCATGTGTGCCAGCGCGAAGTGGCGCATGCGCATAGCGTGCCGCG
CATCACCAGCGCGATCGGCAGCGATCAGGATTGAGAATTCCTCGAG

[a] Underlined sequences indicate inserted restriction sites.

Figure S6: Gene sequences of novel genetically-encoded photosensitizers. The sequences of the *sopp3* (**a**), the *killerorange* (**b**) and the *supernova* gene (**c**) are shown, which were used for the construction of the corresponding expression vectors. The gene sequences were codon optimized for expression in *E. coli* and corresponding genes were synthetically produced.

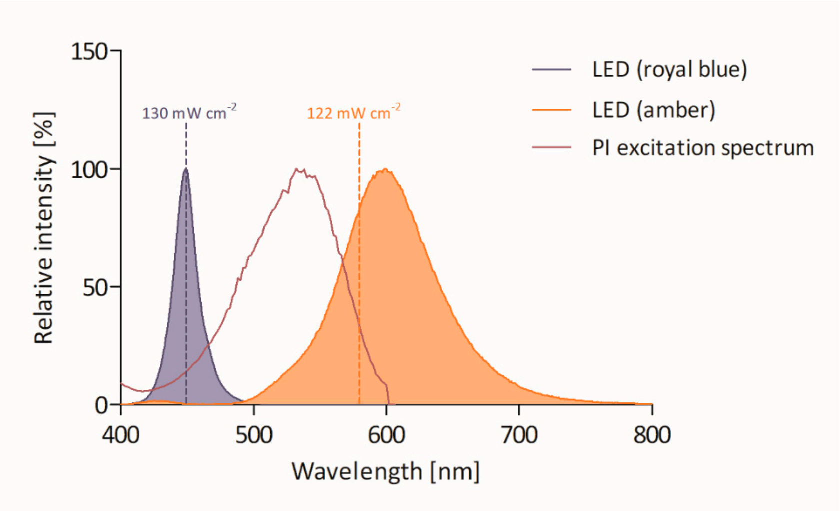

Figure S7: Emission spectra of blue and orange light-emitting high-power LEDs. The emission spectrum of a blue light-emitting LED (royal blue) shows a maximum at 448 nm. The orange light-emitting high-power LED (amber) has an emission maximum at 600 nm. The spectra were measured using a fluorescence spectrometer (Varian Cary Eclipse, Agilent Technologies, Ratingen, Germany). The dashed lines indicate the determined light intensities at the absorption maxima of the used PSs. To estimate the effect of PI-mediated absorption on the LED-mediated excitation of the PSs, the PI excitation spectrum is additionally shown (red line). The spectrum of PI (0.1 mg mL^{-1} salmon sperm in PBS buffer supplemented with 5 μM propidium iodide) was measured at an emission wavelength of 617 nm using a fluorescence spectrometer (Varian Cary Eclipse, Agilent Technologies, Ratingen, Germany. At the blue (450nm) and orange (600nm) emission maximum of the used LEDs, the absorption of PI is rather low and has almost equal and thus negligible effects on the excitation of the tested PSs.

Bacterial strains and plasmids

All bacterial strains, plasmids and oligonucleotides used in this study are listed in Table S1.

Table S1: Bacterial strains, plasmids and oligonucleotides used in this study.

[a] Underlined sequences indicate inserted restriction, mutation or homologous sites.

Strains, plasmids, oligonucleotides	Relevant features, description or sequences[a]	References
**Strains**		
C. glutamicum	Wild-type	[5]
E. coli DH5α	*F^-Φ80lacZΔM15 Δ(lacZYA-argF) U169 recA1 endA1 hsdR17 phoA supE44 thi-1 gyrA96 relA1 deoR*	[6]
E. coli BL21(DE3)	*F^- ompT gal dcm lon hsdSB(rB^- mB^-)λ(DE3 [lacI lacUV5-T7 gene 1 ind1 sam7 nin5])*	[7]
E. coli Tuner(DE3)	*F^- ompT gal dcm lon hsdSB (rB^- mB^-) lacY1(DE3)*	Novagen
P. putida KT2440	Wild-type, recipient for conjugational plasmid transfer	[8]
P. aeruginosa PAO1	Wild-type	[9]
S. aureus	Wild-type	[10]
S. epidermidis	Wild-type	[11]
**Plasmids**		
pET28a(+)	P_{T7}, His6-Tag, MCS, *lacI*, *bla*, Km^R, pBR322 ori, f1 ori	Novagen
pET28a-RBS	P_{T7}, *aphII*, *lacI*, T7 Primer, Km^R	unpublished (Wingen)
pET28a-EcFbFP	*ecfbfp* controlled by the inducible P_{T7}; includes sequence for N-terminal His6-tag; Km^R	[12]
pET28a-Pp1FbFP	*pp1fbfp* controlled by the inducible P_{T7}; includes sequence for N-terminal His6-tag; Km^R	[12]
pET28a-Pp2FbFP	*pp2fbfp* controlled by the inducible P_{T7}; includes sequence for N-terminal His6-tag; Km^R	[12]
pET28a-DsFbFP M49I	*dsfbfp M49I* controlled by the inducible P_{T7}; includes sequence for N-terminal His6-tag; Km^R	[1]
pET28a-SOPP3	*sopp3* controlled by the inducible P_{T7}; includes sequence for N-terminal His6-tag; Km^R	This work
pET28a-KillerOrange	*killerorange* controlled by the inducible P_{T7}; includes sequence for N-terminal His6-tag; Km^R	This work
pET28a-SuperNova	*supernova* controlled by the inducible P_{T7}; includes sequence for N-terminal His6-tag; Km^R	This work
pURE-DsFbFP M49I-LecB	*dsfbfp M49I* – *lecB* fusion, includes sequence for expression by the inducible P_{T7}; N-terminal His6-tag; Amp^R	This work
**Oligonucleotides**		
1 IF_DsFbFPM49I_fow	Binds at the 5′ end of the *dsfbfp M49I* gene, contains homologous ends for InFusion® Cloning. 5′-<u>AGGAGATATACC</u>ATGCGCAGACA TTATCGCGACCTGATAC-3′	This work

2<br>IF_DsFbFPM49I_rev	Binds at the 3′ end of the *dsfbfp m49i* gene, contains homologous ends for InFusion® Cloning and deletes stop codon of *dsfbfp m49i*.<br>5′-CGTCGTCGTCCTCGAAGACCGGGTT CTGGGCGC-3′	This work
3<br>IF_pURE_His_DsFbFP M49I_fo	Binds at the 5′ end of *dsfbfp m49i* gene on pURE DsFbFP M49I plasmid, contains His_6-Tag.<br>5′-ACCACCACCACCACCACATGCGC AGACATTATCGC-3′	This work
4<br>IF_pURE_His_DsFbFP M49I_re	Binds at the 5′ end of *dsfbfp m49i* gene on pURE DsFbFP M49I plasmid, contains His_6-Tag.<br>5′-GGTGGTGGTGGTGGTGCATGGTATA TCTCCTTCTTAAAG-3′	This work

References

1. Endres, S.; Wingen, M.; Torra, J.; Ruiz-González, R.; Polen, T.; Bosio, G.; Bitzenhofer, N.L.; Hilgers, F.; Gensch, T.; Nonell, S.; *et al*. An optogenetic toolbox of LOV-based photosensitizers for light-driven killing of bacteria. *Sci. Rep.* **2018**, *8*, 15021.
2. Westberg, M.; Bregnhøj, M.; Etzerodt, M.; Ogilby, P.R. No Photon Wasted: An Efficient and Selective Singlet Oxygen Photosensitizing Protein. *J. Phys. Chem. B* **2017**, *121*, 9366–9371.
3. Takemoto, K.; Matsuda, T.; Sakai, N.; Fu, D.; Noda, M.; Uchiyama, S.; Kotera, I.; Arai, Y.; Horiuchi, M.; Fukui, K.; et al. SuperNova, a monomeric photosensitizing fluorescent protein for chromophore-assisted light inactivation. *Sci. Rep.* **2013**, *3*, 2629.
4. Sarkisyan, K.S.; Zlobovskaya, O.A.; Gorbachev, D.A.; Bozhanova, N.G.; Sharonov, G. V.; Staroverov, D.B.; Egorov, E.S.; Ryabova, A. V.; Solntsev, K.M.; Mishin, A.S.; *et al*. KillerOrange, a Genetically Encoded Photosensitizer Activated by Blue and Green Light. *PLoS One* **2015**, *10*, e0145287.
5. Abe, S.; Takayama, K.-I.; Kinoshita, S. Taxonomical Studies on Glutamic acid-producing Bacteria. *J. Gen. Appl. Microbiol.* **1967**, *13*, 279–301.
6. Hanahan, D. Studies on transformation of *Escherichia coli* with plasmids. *J. Mol. Biol.* **1983**, *166*, 557–580.
7. Studier, F.W.; Moffatt, B.A. Use of bacteriophage T7 RNA polymerase to direct selective high-level expression of cloned genes. *J. Mol. Biol.* **1986**, *189*, 113–130.
8. Bagdasarian, M.; Lurz, R.; Rückert, B.; Franklin, F.C.; Bagdasarian, M.M.; Frey, J.; Timmis, K.N. Specific-purpose plasmid cloning vectors. II. Broad host range, high copy number, RSF1010-derived vectors, and a host-vector system for gene cloning in *Pseudomonas*. *Gene* **1981**, *16*, 237–247.
9. Holloway, B.W.; Krishnapillai, V.; Morgan, a F. Chromosomal genetics of *Pseudomonas*. *Microbiol. Rev.* **1979**, *43*, 73–102.
10. Treangen, T.J.; Maybank, R.A.; Enke, S.; Friss, M.B.; Diviak, L.F.; Karaolis, D.K.R.; Koren, S.; Ondov, B.; Phillippy, A.M.; Bergman, N.H.; *et al*. Complete Genome Sequence of the Quality Control Strain *Staphylococcus aureus* subsp. *aureus* ATCC 25923. *Genome Announc.* **2014**, *2*, 25923.
11. MacLea, K.S.; Trachtenberg, A.M. Complete Genome Sequence of *Staphylococcus epidermidis* ATCC 12228 Chromosome and Plasmids, Generated by Long-Read Sequencing. *Genome Announc.* **2017**, *5*, 4–5.
12. Wingen, M.; Potzkei, J.; Endres, S.; Casini, G.; Rupprecht, C.; Fahlke, C.; Krauss, U.; Jaeger, K.-E.; Drepper, T.; Gensch, T. The photophysics of LOV-based fluorescent proteins – new tools for cell biology. *Photochem. Photobiol. Sci.* **2014**, *13*, 875–883.
